# When an Implantable Cardioverter Defibrillator can Kill your Patient!

**DOI:** 10.21470/1678-9741-2019-0219

**Published:** 2021

**Authors:** Saulo da Costa Pereira Fontoura, Tiago Luiz Silvestrini, Karila Scardueli Luciano, Gustavo Henrique Sumnienski Bertoldi, Eliana Costa Pelissari, Rafael de March Ronsoni

**Affiliations:** 1 Instituto de Ritmologia Cardíaca, Santa Catarina, Brazil.; 2 Serviço de Eletrofisiologia e Arritmia Cardíaca, Centro Hospitalar UNIMED, Santa Catarina, Brazil.; 3 Universidade da Região de Joinville - UNIVILLE, Santa Catarina, Brazil.; 4 Serviço de Cardiologia Pediátrica, Hospital Infantil Dr. Jesser Amarante Faria, Santa Catarina, Brazil.

**Keywords:** Ventricular Fibrilation, Defibrillations, Implantable, Syncope, Arrhythmias, Cardiac, Primary Prevention, Shock, Emergency Service, Hospital

## Abstract

Inappropriate therapy due to noise oversensing caused a true ventricular fibrillation (VF) and a life-threatening event in a patient. A 19-year-old patient with surgically corrected congenital heart disease and systolic dysfunction had an implantable cardioverter defibrillator implanted for primary prevention in 2013. This patient was admitted at the Emergency Department in June 2018 after receiving eight shocks from the device on the same day, with a prolonged syncope after the third shock. Another noise-induced VF detection occurred, and two inappropriate shocks followed sequentially, causing true VF. Four appropriate shocks were subsequently needed until sinus rhythm was finally restored.

**Table:** 

Abbreviations, acronyms & symbols	
ICD	= Implantable cardioverter defibrillator
VF	= Ventricular fibrillation
VT	= Ventricular tachycardia

## INTRODUCTION

Implantable cardioverter defibrillator (ICD) therapy is a fundamental tool in the prevention of sudden cardiac death. Despite this, it may also carry potentially serious risks. In the following section, we report a life-threatening event caused by a device malfunction.

## CASE REPORT

Our patient is a 19-year-old Caucasian male with a Biotronik Lumax 740 DR-T ICD, with Solia S53 ProMRI and Linox Smart ProMRI leads, implanted in 2013 for primary prevention of sudden death due to a complex congenital cardiac disease. The disease under discussion is a pulmonary atresia associated with interventricular communication and dextrocardia. A complete surgical correction was performed when the patient was three years old. Important ventricular dysfunction ensued during the following years and a severe mitral regurgitation was also diagnosed. He underwent a mitral valvuloplasty when he was nine years old. Despite these efforts, his left ventricular ejection fraction was 30% at the time it was decided by another team of specialists for the implantation of the ICD to perform primary prevention.

The device settings at the time of the described events were the following: DDD mode, base rate 50 ppm. Ventricular arrhythmia detection was programmed in three different zones, starting at 330 ms for ventricular tachycardia (VT) 1, 300 ms for VT 2, and 280 ms for ventricular fibrillation (VF). Antitachycardia therapy for VT 1 zone was off. For VT 2, it was two times bursts, then two times ramp, and then sequential 40 Joules shocks. In VF zone, the device would deliver burst while charging, and then sequential 40 Joules shocks. Detection parameters were set for 26 beatings for VT 1, 18 for VT 2, and 10 out of 14 for VF. The onset, stability, and SMART detection algorithms were turned on for VT 1 and VT 2.

The patient was admitted at the Emergency Department in June 2018 after receiving eight shocks from the device on the same day, with a prolonged syncope after the third shock. This patient had not received ICD therapy since implantation until that day. Upon interrogation, there were twelve episodes identified by the ICD algorithm as VF, four of them had the VF treatment protocol aborted because of “termination” of the episode, and the remaining eight were treated with shocks (ventricular impedance 564 ohms and shock impedance 40 ohms - both are stable).

The first episodes identified as VF by the device were clearly caused by inappropriate detection of arrhythmia due to periods of noise detection in the ventricular lead, some of which were too short, so shock therapy was cancelled before being delivered. One episode of lead noise identified as VF was long enough to trigger an inappropriate shock, followed by a reduction in the lead noise and “termination” of the episode.

**Fig. 1:**
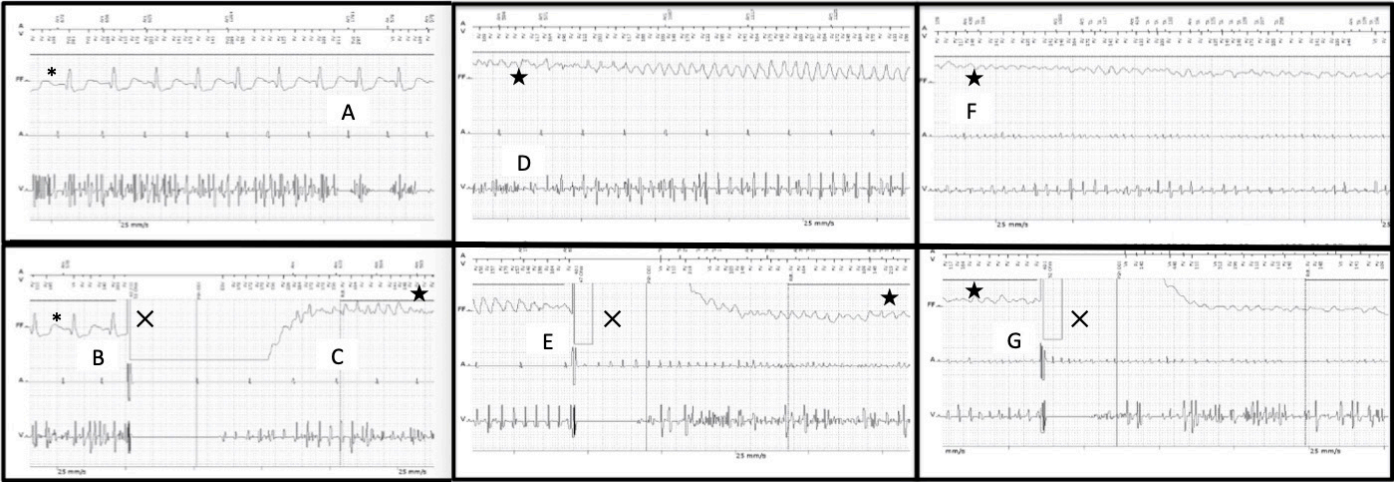
Inappropriate detection of ventricular fibrillation due to noise in the ventricular lead (A), followed by inappropriate shock therapy (B), and implantable cardioverter defibrillator-induced ventricular fibrillation (C). Ventricular fibrillation (D and F) and unsuccessful shock therapy (two episodes - E and G). * sinus rhythm; X shock therapy; ★ ventricular fibrillation.

Another noise-induced VF detection occurred, and two more inappropriate shocks followed sequentially, causing true VF, as seen in Figure 1.

**Fig. 2:**
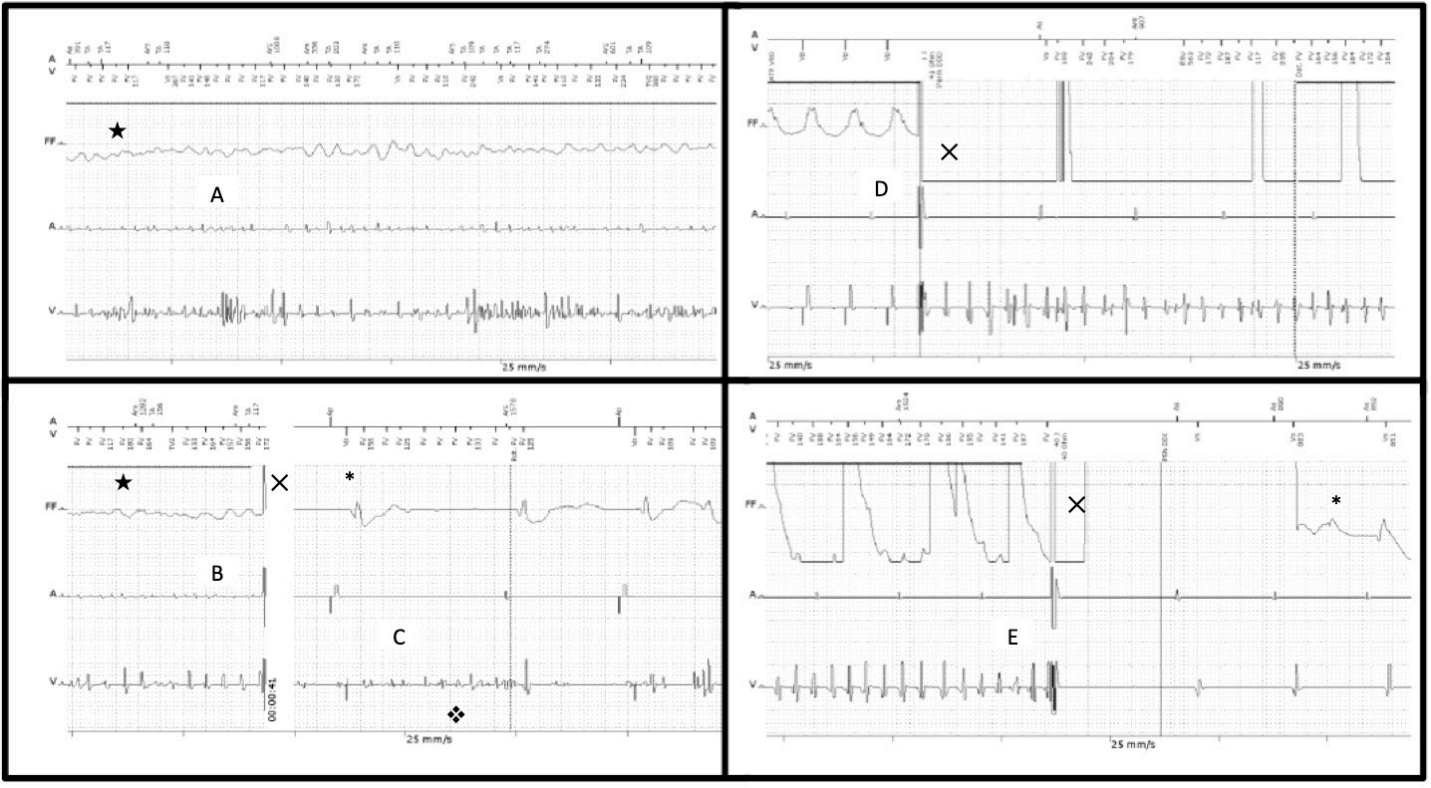
(A) Ventricular fibrillation (VF). Fourth appropriate shock finally terminated the arrhythmia (B and C). Notice that the noise remained in the ventricular lead after sinus rhythm restoration (C). A new implantable cardioverter defibrillator-induced VF (t wave shock) (D) would occur later on the defibrillation test (40 Joules) (D) (see Case Report for further details). * sinus rhythm; X shock therapy; ★ ventricular fibrillation; vnoise in the ventricular lead.

Four appropriate shocks were subsequently needed until sinus rhythm was finally restored, as seen on Figures 2 A, B, and C.

Upon interrogation, these episodes were retrieved and analyzed. Pacing threshold and sensibility were stable and similar to previous readings, while ventricular lead impedance rose and shock impedance fell, both still within normal ranges.

Once the diagnoses of inappropriate VF detection caused by noise and of inappropriate shocks causing true VF were made, the ICD therapy was shut down temporarily and the patient was transferred to the intensive care unit for observation. A new shock lead implantation in the right ventricle was indicated and successfully performed through cephalic vein dissection and effective defibrillation testing with 40 Joules (Figures 2 D and E). No attempt of extraction of the old malfunctioning lead was made, on account of the patient's anatomical complexity. Since the surgery, he has not received any further appropriate or inappropriate shocks.

## DISCUSSION

Inappropriate ICD shock incidence in congenital heart disease ranges from 20% to 25%. The most common causes are supraventricular tachycardias, followed by other electrogram noise, and T wave oversensing^[1]^. Many of the new algorithms aim to lower inappropriate shocks by improving discrimination between supraventricular and ventricular rhythms, and not so much by recognizing electrogram interference. Those algorithms vary depending on the manufacturer, but the fundamental principles used by most of them are the same. One such principle is the analysis of the tachycardia cycle characteristics, such as the RR cycle compared to the PP cycle, RR stability, the PR association, and so on. For example, RR cycle smaller than PP cycle is compatible with VT. Another principle is the tachycardia onset, which averages the last few RR cycles and compares it with the following few RR cycles. An abrupt change in cycle length favors the diagnosis of VT. Finally, the ventricular signal morphology of the tachycardia can be compared to the base ventricular signal to help with this differential diagnosis. All devices provide warning alerts for lead failure when it is associated with an abrupt change in lead parameters such as impedance readings. There are also algorithms to provide alerts when ventricular cycle lengths are nonphysiologically short, suggesting oversensing. When signals are sensed on the intracardiac electrogram and not the shock electrode, there can be alerts, and in some cases, withholding of therapy, which again should be used with caution^[2]^.

The explanation for noise oversensing is the insulation defect of the ventricular lead, which was demonstrated by the event recording as prolonged and repeated episodes of noise. Oversensing is also possible without structural lead failure^[3]^.

In this case, we theorize it was a lead failure, perhaps insulation breaches, which caused oversensing, rather than an external source, since inappropriate detections occurred in more than one place, even at the hospital, and this patient has had no new episode since the electrode replacement. The original electrode, however, wasn't extracted for confirmation of this hypothesis.

Previous studies have described an association between inappropriate shocks and higher mortality, though a more recent Danish register has not reported a difference in mortality^[4]^. In this case, however, the risk of death was clearly high as not only VF was induced, but multiple shocks were needed to terminate the arrhythmia. Others have shown VF induced by inappropriate therapy and followed by a life-threatening or even fatal outcome^[5,6]^, which are reminders of the danger posed by such episodes.

This case illustrates the perils associated with inappropriate shocks and reinforces the importance of avoiding these events. Appropriate indication for the implantation of ICDs and the correct use of algorithms for a safe programming of the device are paramount to achieve this goal. Further strategies would be desirable to avoid conditions that cause lead electrogram interference or to improve device discrimination between electrogram interference and true ventricular arrhythmias.

**Table:** 

Authors' roles & responsibilities			
SCPF	Substantial contributions to the conception or design of the work; or the acquisition, analysis, or interpretation of data for the work; drafting the work or revising it critically for important intellectual content; agreement to be accountable for all aspects of the work in ensuring that questions related to the accuracy or integrity of any part of the work are appropriately investigated and resolved; final approval of the version to be published	GHSB	Substantial contributions to the conception or design of the work; or the acquisition, analysis, or interpretation of data for the work; drafting the work or revising it critically for important intellectual content; agreement to be accountable for all aspects of the work in ensuring that questions related to the accuracy or integrity of any part of the work are appropriately investigated and resolved; final approval of the version to be published
TLS	Substantial contributions to the conception or design of the work; or the acquisition, analysis, or interpretation of data for the work; drafting the work or revising it critically for important intellectual content; agreement to be accountable for all aspects of the work in ensuring that questions related to the accuracy or integrity of any part of the work are appropriately investigated and resolved; final approval of the version to be published	ECP	Substantial contributions to the conception or design of the work; or the acquisition, analysis, or interpretation of data for the work; drafting the work or revising it critically for important intellectual content; agreement to be accountable for all aspects of the work in ensuring that questions related to the accuracy or integrity of any part of the work are appropriately investigated and resolved; final approval of the version to be published
KSL	Substantial contributions to the conception or design of the work; or the acquisition, analysis, or interpretation of data for the work; drafting the work or revising it critically for important intellectual content; agreement to be accountable for all aspects of the work in ensuring that questions related to the accuracy or integrity of any part of the work are appropriately investigated and resolved; final approval of the version to be published	RMR	Substantial contributions to the conception or design of the work; or the acquisition, analysis, or interpretation of data for the work; drafting the work or revising it critically for important intellectual content; agreement to be accountable for all aspects of the work in ensuring that questions related to the accuracy or integrity of any part of the work are appropriately investigated and resolved; final approval of the version to be published
